# Assessing rigid modes of thinking in self-declared abortion ideology: natural language processing insights from an online pilot qualitative study on abortion attitudes

**DOI:** 10.1186/s40814-022-01078-0

**Published:** 2022-06-16

**Authors:** Danny Valdez, Kristen N. Jozkowski, Katherine Haus, Marijn ten Thij, Brandon L. Crawford, María S. Montenegro, Wen-Juo Lo, Ronna C. Turner, Johan Bollen

**Affiliations:** 1 Indiana University School of Public Health, 1025 E 7th Street, Bloomington, IN 47405 USA; 2 Department of Data Science and Knowledge Engineering, Universiteit Maastricht, P.O. Box 616, 6200 MD Maastricht, Netherlands; 3 Indiana University College of Arts and Sciences, 107 S Indiana Ave, Bloomington, IN 47405 USA; 4 University of Arkansas, 1 University of Arkansas, Fayetteville, AR 72701 USA; 5 Luddy School of Informatics, Computing and Engineering, 919 E. 10th St., Bloomington, IN 47408 USA

**Keywords:** Abortion, Qualitative, Natural language processing, Psychology, Cognitive distortions

## Abstract

**Introduction:**

Although much work has been done on US abortion ideology, less is known relative to the psychological processes that distinguish personal abortion beliefs or how those beliefs are communicated to others. As part of a forthcoming probability-based sampling designed study on US abortion climate, we piloted a study with a controlled sample to determine whether psychological indicators guiding abortion beliefs can be meaningfully extracted from qualitative interviews using natural language processing (NLP) substring matching. Of particular interest to this study is the presence of cognitive distortions—markers of rigid thinking—spoken during interviews and how cognitive distortion frequency may be tied to rigid, or firm, abortion beliefs.

**Methods:**

We ran qualitative interview transcripts against two lexicons. The first lexicon, the cognitive distortion schemata (CDS), was applied to identify cognitive distortion n-grams (a series of words) embedded within the qualitative interviews. The second lexicon, the Linguistic Inquiry Word Count (LIWC), was applied to extract other psychological indicators, including the degrees of (1) analytic thinking, (2) emotional reasoning, (3) authenticity, and (4) clout.

**Results:**

People with polarized abortion views (i.e., strongly supportive of or opposed to abortion) had the highest observed usage of CDS n-grams, scored highest on authenticity, and lowest on analytic thinking. By contrast, people with moderate or uncertain abortion views (i.e., people holding more complex or nuanced views of abortion) spoke with the least CDS n-grams and scored slightly higher on analytic thinking.

**Discussion and conclusion:**

Our findings suggest people communicate about abortion differently depending on their personal abortion ideology. Those with strong abortion views may be more likely to communicate with authoritative words and patterns of words indicative of cognitive distortions—or limited complexity in belief systems. Those with moderate views are more likely to speak in conflicting terms and patterns of words that are flexible and open to change—or high complexity in belief systems. These findings suggest it is possible to extract psychological indicators with NLP from qualitative interviews about abortion. Findings from this study will help refine our protocol ahead of full-study launch.

## Key messages regarding feasibility

- We were uncertain whether words indicative of rigid thinking could be meaningfully extracted from qualitative interviews about personal abortion beliefs and ideology using natural language processing methods.
- As a pilot investigation for a forthcoming probability-based sampling study, we conducted 20 interviews with a convenience sample of US adults.
- Using two validated lexicons, we determined that it was possible to extract indicators of rigid thinking from qualitative interviews about abortion ideology, including differences in rigidity based on one’s personal abortion views (i.e., strongly pro-choice/pro-life versus equally pro-choice and pro-life).
- Identified differences within our limited sample suggest this approach can be leveraged with the larger sample to afford meaningful comparisons between groups, including gender, race, political affiliation, and others.

## Background

Large segments of the US population identify as either pro-choice (broadly defined as having more favorable views regarding abortion access) or pro-life (broadly defined as having less favorable views regarding abortion access) [1]—highly politicized terms that frame abortion as part of a political movement. Yet, despite the wide association of these labels as positions on abortion, numerous studies have shown that abortion beliefs are not similarly dichotomous or static but rather contextual, complex, at times contradictory, and can evolve over time [2–5].

Although research has examined the complexity and nuances of abortion views, less is known about the psychological processes through which personal ideologies about abortion are constructed. For example, beyond determining whether someone’s abortion views are complex (e.g., conflicted, uncertain, or contradictory) or polar (i.e., identifying in extreme positions such as strongly pro-choice or pro-life or strongly supportive of or opposed to abortion), questions remain about the psychological processes through which people arrive at their abortion beliefs and how such beliefs are communicated to others.

The construction and processing of beliefs about the world and oneself is a growing research area and remains a crucial element of cognitive behavioral therapy (CBT). A core component of CBT is focused on identifying and addressing cognitive distortions in spoken language. A cognitive distortion is a specific pattern of words and/or thoughts through which people view themselves and the world in overly rigid and absolute ways [6]. There are several cognitive distortion categories such as “all-or-nothing reasoning,” “labeling and mislabeling,” “jumping to conclusions,” and “overgeneralization.” Indeed, many typologies of cognitive distortions distinguish at least 12 distinct categories, including those mentioned above [6]. Research shows that the presence of cognitive distortions in daily language may be indicative of increased risk of affective disorders (e.g., anxiety, depression) [7], emotionally biased thinking [8], and importantly for our purposes, one-dimensional, absolutist perspectives [9]. Given that cognitive distortions mark rigid thinking patterns, they may be associated with political and societal polarization [10]. By extension, cognitive distortions may be relevant to studying how people establish beliefs about abortion and how strongly those with ardent, polarized abortion views argue in favor of and adhere to those beliefs. However, these typologies are intended as descriptive summarizations, not to make claims about the cognitive structure of cognitive distortions per se. In fact, most, if not all, cognitive distortions may generally fall under the broader moniker of rigid modes of thought.

Though conventionally framed in the context of CBT, the notion of cognitive distortions and their expression in language are now increasingly applied to study other facets of communication [11] using natural language processing (NLP) and machine learning methods. Computational advances leveraging NLP and machine learning have made it possible to detect markers of cognitive distortions in text data. This development is primarily enabled by NLP methods which analyze language to infer a range of psychosocial states and phenomena from individual or group language [12, 13]. NLP is often used with electronic text sources such as social media [13, 14] to examine a variety of sociolinguistic and psychological phenomena, including mental health and substance use [13], broad social concerns such as the ongoing COVID-19 pandemic [15–17], and estimating suicide risk [18].

Beyond exploratory NLP applications, these methods have also been leveraged to identify opinions by mining for specifically valanced words to determine whether people express positive or negative affect about certain topics [19]. Similar techniques have been applied to detect cognitive distortions in written language, for example, to construct digital profiles of social media users with internalizing disorders (e.g., anxiety and depression), demonstrating that the online language of people with depression has significantly higher rates of markers of cognitive distortions [20]. However, such methods have not been applied as readily in the examination of contentious social issues. Given that abortion has been and remains a particularly salient and contentious social issue in the US, it would be helpful to understand if and how cognitive distortions may manifest when people talk about abortion in qualitative interviews.

Here we examine the psychological processes by which abortion beliefs are grounded based on the prevalence of cognitive distortions. We use an NLP framework to analyze a small sample of qualitative interviews and detect the presence of cognitive distortion n-grams (sequences of adjacent words) as indicators of rigid thinking about abortion. Thus, we assessed the degree to which cognitive distortions may play a role in shaping abortion beliefs. Given this novel application, we were also interested in assessing the feasibility of using NLP and lexical tracking when examining cognitive distortions in people’s discussions of abortion.

Two research questions guided this study:

- RQ1: Can we distinguish thinking and communicative differences in personal ideologies across the abortion belief spectrum?
- RQ2: How can NLP be leveraged to study patterns of thought in qualitative data about abortion?

Given that cognitive distortions are generally associated with thinking patterns considered overly rigid, dichotomized, or absolutist, we hypothesize that a high prevalence of cognitive distortions in language may be indicative of unwavering or firm abortion beliefs and attitudes. In contrast, lower cognitive distortion prevalence may be indicative of more flexible, less polarized (i.e., more complex) abortion views. Thus, by applying NLP methods to uncover latent psychological indicators within qualitative data about abortion beliefs, we may be able to advance our understanding of people’s conceptualizations of the complexity of abortion beyond current quantitative/qualitative studies. Indeed, such an approach can infer deeper meaning about the processes contributing to complex and/or noncomplex beliefs. Additionally, insights into the cognitive processes behind belief systems, disregarding abortion ideology (e.g., identifying as pro-life, pro-choice, neither, or both; holding strong attitudes in support of or opposition to abortion), may also add deeper nuance to what is already known about the psychology of abortion attitudes in the US.

## Methods

### Data

This pilot study is part of a national, ongoing investigation of people’s attitudes toward abortion in the US. Potential study participants were recruited via the Growth from Knowledge Panel (GfK) using quota-based sampling techniques to identify a diverse sample of adults residing in the USA. As part of the study’s procedure, participants were initially contacted via email to complete a 20-min online survey on social issues, focusing on abortion. Data collected for this study were used to refine the protocol for a forthcoming national study of abortion attitudes using probability-based sampling. All data procurement adhered to guidelines enforced by the Institutional Review Board (IRB).

### Piloting a qualitative interview protocol

#### Survey creation and interview protocol development

Our larger study evaluates US abortion attitudes with a nationally representative sample of US adults across two phases; the present study represents a pilot for these procedures. The first stage involves inviting a panel of participants to complete a 20-min survey on social issues with an emphasis on abortion. The second stage involves in-depth qualitative interviews to examine the extent of a person’s complex abortion views. The goal of the pilot study, performed ahead of the launch of the larger investigation, was to test the effectiveness of the survey and interview protocol and alter either if necessary.

For phase 1, we designed the survey to measure abortion beliefs regardless of ideology. The survey included a series of abortion-related assessments, which we used to develop an abortion complexity score. These measures included attitudes toward abortion legality and abortion morality, how people identify in terms of abortion labels (e.g., pro-life, pro-choice, neither, both), assessments of people’s views on abortion legislation, and personal engagement with abortion (e.g., whether they have had an abortion, know someone who has an abortion, would help someone pay for or get an abortion). We compiled people’s response patterns on these measures to develop a scoring continuum from people who are most supportive of abortion to those who are most opposed to abortion; those falling in the middle of this theoretical continuum were deemed to hold “complex” views toward abortion [21]. Because we were most interested in examining people with complex perspectives regarding abortion for our in-depth interviews, we targeted our recruitment for interviews from those with survey responses that indicated “complexity” based on this scoring [5, 21].

In accordance with best practices for qualitative research [22], for phase 2, we piloted the interview protocol with a small sample of participants, which represents the present study. For this feasibility study, we aimed to test three preliminary facets of the project: (1) the survey’s ability to identify people with complex abortion views, (2) the effectiveness of the interview protocol as a tool for capturing abortion attitude complexity, and (3) the interviewers’ collective ability to use the survey and interview protocol as intended. Data collected for the pilot study were used to refine the survey and interview protocols ahead of the larger study. Please note that these pilot data are in no way intended to make statements about the abortion climate in the USA but are used strictly to evaluate the merit of the protocol and test proposed analyses.

Between August and November of 2020, we administered our survey to people comprising GfK’s national panel. The initial sample included 1583 participants who completed the online survey and met quota requirements^1^ and requirements set forth by GfK for sufficient quality data^2^. Of these participants, we contacted 88 individuals who had complex abortion scores and agreed to participate in a one-on-one pilot interview.

From those initially contacted, 16 people responded and completed the interview. We attribute the low response rate to scheduling conflicts, no-responses, no-shows, and noneffective recruitment strategies. For example, we initially contacted participants via email for participation in the follow-up interviews but realized this tactic was ineffective. As such, we modified our procedures to recruit via text message, which yielded a much better response from participants.

Given the limited time between the pilot study and the launch of the larger investigation, we deemed these 16 sufficient to test the protocol for several reasons. First, this sample size would allow each interviewer to conduct two practice interviews. Second, 16 completed interviews account for approximately 10% of the proposed sample for the larger study (*n* = 170). Third, a more intimate sample would allow us to review each interview carefully, practice proposed analyses, provide feedback to the interviewer, and more appropriately see alterations that needed to be made.

We conducted the pilot interviews in two phases. The first phase of our piloting process tested the first draft of the interview protocol among an initial sample of *n* = 6 participants (one interview per interviewer). We used these initial interviews to train team members in the interview process and determine if alternations to our protocol were required. Upon completion of the initial interviews, we evaluated the quality of the data and sought feedback from qualitative interview experts on our team. We determined alterations to the protocol were needed to streamline the interview process (e.g., shorter the length of the interview protocol) and add additional clarity (i.e., adding question blocks to elicit specific information on personal abortion beliefs). After we made these revisions, we invited a second cohort of people to participate in the interviews as part of a more-formal pilot study (*n* = 10) (roughly two per interviewer). Additional interviews were conducted in Spanish; however, those data fall beyond the scope of this work.

### Sample

Our pilot interview sample comprised 12 men and 4 women. The mean age of our sample was 49.1 (*SD* = 11.9). Participants were diverse in their self-described abortion beliefs. Additionally, as NLP studies conventionally report the total number of words analyzed as a component of the sample, we analyzed approximately *n* = 135,000 words, to which we applied an n-gram lexical match analysis. Please see Table 1 for a breakdown of the sample by demographic variables.

**Table 1:** Participant demographic information and cognitive distortions spoken per interview

Participant ID	Gender	Age	Abortion identity	Pol affiliation
1	Woman	70	Neither pro-choice nor pro life	Democrat
2	Woman	46	Strongly pro-choice	None
3	Woman	65	Strongly pro-choice	Democrat
4	Man	44	Strongly pro-choice	Libertarian
5	Man	24	Strongly pro-choice	Democrat
6	Man	47	Strongly pro-choice	Democrat
7	Man	58	Slightly pro-choice	Democrat
8	Woman	61	Equally pro-choice and pro-life	Democrat
9	Man	35	Equally pro-choice and pro-life	Democrat
10	Man	35	Equally pro-choice and pro-life	Independent
11	Man	59	Equally pro-choice and pro-life	Republican
12	Man	52	Slightly pro-life	Republican
13	Man	47	Moderately pro-life	Democrat
14	Man	51	Moderately pro-life	Republican
15	Man	43	Moderately pro-life	Democrat
16	Man	49	Strongly pro-life	Democrat

#### Lexical matching

We applied a lexical substring-matching technique to gauge whether our interview texts contain evidence of (1) cognitive distortions, (2) authenticity, (3) clout, (4) analytical thinking, and (5) perceptiveness. As an unsupervised NLP methodology, lexical sub-string matching records the prevalence of terms in the content of a corpus of interest to extract indicators of particular psychological or social constructs from the text [13]. The assumption underlying lexical matching is that the terms used in a person’s language are indicative of their psychological state. Therefore, a computer algorithm can scan text data and tally the occurrences of a set of preselected or rated terms [12] from a lexicon designed to capture such states. For example, we may scan text for terms embedded in an emotion lexicon consisting of words indicative of emotional states, e.g., “happy,” “sad,” and “angry” [22].

**Fig. 1:**
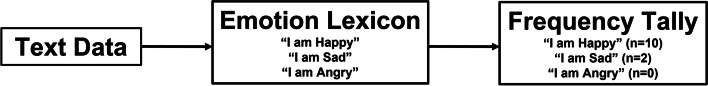
A conceptual diagram of lexicon matching

Lexicons can be constructed to identify diverse phenomena in text data beyond polarity, including affective states, well-being, and other psychological markers [21]. Please refer to Fig. 1 for a sample conceptual explanation of lexical matching. Note that a lexicon may consist of terms that either combine groups of multiple words or more general n-grams of n consecutive words that capture the local structure of language, for example, the 3-gram “I am happy” vs. the 5-gram “I am not very happy.” In general, n-grams (i.e., a series of connected words) are better markers of emotional states than single term queries (i.e., happy or sad).

Lexicons can be unipolar (e.g., “emotionality”) or bipolar (“positive” vs. “negative” sentiment) and can contain words that were rated by human subjects to gauge the degree to which they signify a particular psychological or lexical characteristic. The lexicon of the Valence Aware Dictionary and sEntiment [sic] Reasoner (VADER) sentiment analysis tool, which is commonly used to quantify the degree of positivity/negativity of written language [20], is composed of 7516 English terms that were numerically rated on a scale of −4 to +4 by multiple human raters in terms of their positive vs. negative affect. For example, the average human rating of “murder” and “happy” is respectively −3.7 and +2.7. The presence of the lexicon words in a text can be detected and tallied, and subsequently, their ratings can be used as an indicator of the text’s valence.

This study applied two lexicons to the transcribed qualitative interviews. The first lexicon, hereby referred to as the cognitive distortion schemata (CDS) lexicon, was introduced by Bathina and colleagues [23] to observe the structural and lexical patterns associated with the expression of distorted thinking. The CDS lexicon consists of a list of 214, one to five n-grams (i.e., single words to sequences of 5 words) shown to indicate cognitive distortions. The CDS lexicon is broadly composed of groups of n-grams separated into 12 classes of commonly distinguished cognitive distortion types as identified in validated psychological inventories. Each class of CDS contains about 15–30 phrases and associated variants (see Table 2 for a breakdown of the CDS lexicon). Example categories include labeling and mislabeling—i.e., *ascribing labels to self or others* indicated by n-grams such as “I am a,” “you are a,” and associated contractions (i.e., I’m a, You’re a). Dichotomous reasoning—i.e., *framing issues or events in black and white terms* such as “always” and “never,” and catastrophizing—i.e., *predicting the outcome, usually in negative terms, among others* such as “will fail,” “will never work.” All words and phrases that indicate a cognitive distortion in the CDS lexicon originate from validated scales and other psychological inventories. The CDS lexicon has been empirically tested with large-scale social media data, within- and between-subject data, as well as large historical records of societal language, and validated by a panel of 8 licensed clinical psychologists unaffiliated with this study.

**Table 2:** List and definitions of cognitive distortions (CDS)^a^ and select examples of terms in the CDS^a^ lexicon

Distortion	Definition	Select terms	Example sentence
Labeling and mislabeling	*A way of thinking characterized by how we overgeneralize a trait to the whole person*	I am a	“**I am a** firm believer that abortion is a woman's choice”
		He is a	
		They are a	
Catastrophizing	*A way of thinking characterized by predicting an outcome or jumping to a conclusion that may or may not happen*	…Will go wrong	“If she has an abortion, **it will be terrible** for her family”
		…Will be terrible	
		…Will be a disaster	
Dichotomous reasoning	*A way of thinking characterized by polarized (i.e., black and white) expressions*	Only	“I have **always** felt that abortions are morally wrong”
		Ever	
		Always	
Emotional reasoning	*A way of thinking characterized by drawing conclusions using emotional truth over empirical evidence*	But I feel	“Abortion are legal; **but I feel** they should not be”
		Because I feel	
		Since it feels	
Disqualifying the positive	*A way of thinking characterized by filtering out a positive experience and focusing only on negative aspects*	…Great but	“Abortions are **great but** at what cost?”
		…Acceptable, yet	
		…. Not that good	
Magnification and minimization	*A way of thinking characterized by exaggerating or dismissing key ideas*	Worst	“Criminalizing abortion would be the **worst** decision ever”
		Best	
		No matter	
Mental filtering	*A way of thinking characterized by projecting one's views outward*	All I see	“**All I see** are millions of unborn babies being killed”
		…Can only think	
		If I only	
Mind reading	*A way of thinking characterized by assuming one's personal views mirror others*	Everyone knows	“**Everyone knows** abortion is a public good even if they won't admit it”
		No one believes	
		They all know	
Fortune-telling	*A way of thinking characterized by attempting to predict what is largely unknown*	I will not…	“**They will not** outlaw abortion outright, there's just no way!”
		We will not…	
		He/she will…	
Overgeneralizing	*A way of thinking characterized by viewing a single event as an invariable rule*	Completely	“Regretting an abortion? It will **always happen**!”
		Always happens	
		Every single time	
Personalizing	*A way of thinking characterized by placing blame entirely on oneself even if disconnected from the event*	All me	“I tend to not have many friends **because of my** liberal abortion views”
		Because of my	
		My responsibility	
Normative statements	*A way of thinking characterized by projecting one's views as correct or right*	Should [not]	“We **should be** honoring a baby's right to life!”
		Ought [not]	
		Must	

^a^CDS is an acronym for the cognitive distortion schemata. Please also note terms listed per CDS are non-exhaustive

The second lexicon used for this study, the Linguistic and Inquiry Word Count (LIWC), is a highly validated word-processing engine that evaluates the presence of words in a text that marks psychological states [24]. LIWC is a gold standard for text mining in the psychological and social sciences [25, 26] and can be used to mine text for several psychological indicators, including authenticity—i.e., *speaking openly and truthfully*; perceptiveness—i.e., *speaking in terms of feelings or emotion*; analytic thinking—i.e., *the degree to which people use words indicative of higher-order thinking*; and clout—i.e., *the ability to speak about something with authority*. For insight into the LIWC lexicon, associated variables, and validation processes, please refer to https://liwc.wpengine.com/interpreting-liwc-output/. See Table 3.

**Table 3:** Breakdown of included LIWC^a^ variables (2015 Dictionary)

Indicator	Definition	Select terms	Example sentence
Authenticity	*The degree to which someone speaks or writes in language that is open, honest, and truthful*	***Proprietary***	‘Please be aware that the views about abortion I am expressing are **entirely my own**”
Clout	*The degree to which someone speaks or writes in language that is indicative of authority*	***Proprietary***	“**I am a nurse so I know quite a bit** about abortion”
Analytic thinking	*The degree to which someone speaks or writes in language that is indicative of higher-order thinking*	***Proprietary***	“**There are many reasons why someone may choose to have an abortion** and they are complex”
Perceptiveness	*The degree to which someone speaks or writes in language that is indicative of feelings or emotions*	I see…	“**I feel** as though abortion is an incredibly traumatic experience even though I've never experienced one”
		I hear…	
		I feel…	

^a^The Linguistic Inquiry and Word Count (LIWC) is a proprietary algorithm and does not disclose the precise words used to calculate authenticity, clout, and analytic thinking scores. However, a detailed description of each variable and how the variable was calibrated can be found at https://liwc.wpengine.com/interpreting-liwc-output/

#### Procedure

Our initial screener survey was administered to a national panel of participants in the USA via GfK, an online sample aggregator. Incentivized inventions were sent to eligible participants. After participants completed the survey, they were asked if they were interested in participating in a follow-up interview. The research team contacted interested participants who were deemed to have complex attitudes toward abortion (see [21]) to schedule a follow-up interview. Interviews were semi-structured, video and/or audio recorded, and lasted approximately 1 h. Interviewers followed a consistent interview protocol that comprised lead-off questions. Based on participants’ responses to these questions, interviewers followed up accordingly to glean information about participants’ thoughts and beliefs regarding abortion as well as factors that underlie these thoughts and beliefs. In appreciation of their time, participants received a US $100 gift card. All interviews were transcribed verbatim.

#### Analysis

Two researchers independently reviewed interview transcripts with the original audio file for clarity and accuracy. After completing this quality check, we then edited each transcript to only contain portions of the interview that originated from the participant. Portions spoken by the interviewer were removed from each transcript to ensure that language captured by the CDS and LIWC lexicons originated from the participant and not another party. Each interview was saved as a separate CSV file and stored in one folder for further analysis.

We next performed two independent lexical matching analyses. The first analysis compared the qualitative transcripts against the CDS lexicon to detect cognitive distortions. This analysis aimed to tally the total number of CDS spoken during each participant’s interview. Higher numbers denoted greater evidence of distorted/rigid thinking, and lower numbers indicated greater evidence of impartial or unbiased thinking. Next, we calculated the sum score of the total number of CDS uttered per interview. We standardized our sum scores by dividing each score by the number of minutes it took to complete the interview (e.g., 200 CDS/60 min = 3.33 CDS per minute). We then reran our data through the LIWC lexicon, specifically testing the following variables: authenticity, analytics, perception, and clout. For this analysis, the LIWC lexicon identified the total percentage of words that capture each mentioned component. LIWC displays results as percentages. Thus, if someone were to score 15.7 on authenticity, we could infer that approximately 16% of words in the interview indicated the person was speaking in an authentic manner. For more insight into benchmarks and interpreting LIWC output, see Pennebaker et al. (2015).

## Results

This study examined the psychological mechanics by which abortion beliefs are grounded. We used two validated lexicon analysis tools to mine a series of pilot qualitative interviews for indicators of rigid abortion thinking. We identified several patterns and indicators of rigidity for personal abortion beliefs. We present those findings below without comment.

### CDS summary

First, we observed variability in total cognitive distortions spoken during each interview. Table 4 provides the breakdown of the total number of CDS spoken during each interview and the number of CDS per minute. The average number of total spoken CDS per interview was 182.12 (*SD* = 81.61). The average number of CDS per minute (derived by dividing the total number of CDS by interview length) was 2.94 (*SD* = 1.49). Table 5 displays the frequency of CDS classes. The most represented categories of CDS include mind reading (42%; i.e., *believing others’ views mirror their own*); dichotomous reasoning (30%; i.e., *one-dimensional thinking*); and normative thinking (15%; i.e., *projecting one’s views as correct*). The remaining classes of CDS were represented at a minimal capacity (see Table 5). We also observed that women had higher CDS per minute than men (women = 3.35 versus men = 2.80). Other similar demographic comparisons yielded marginal to almost no differences.

**Table 4:** Participant ID and CDS^a^ spoken per interview

Participant ID	CDS total	CDS per minute	Abortion Identity
1	122	1.91	Neither pro-choice nor pro-life
2	308	4.46	Strongly pro-choice
3	90	1.38	Strongly pro-choice
4	226	5.65	Strongly pro-choice
5	152	4.22	Strongly pro-choice
6	327	5.19	Strongly pro-choice
7	259	4.80	Strongly pro-choice
8	144	1.92	Equally pro-choice and pro-life
9	122	1.85	Equally pro-choice and pro-life
10	130	1.66	Equally pro-choice and pro-life
11	236	2.95	Equally pro-choice and pro-life
12	126	2.26	Slightly pro-life
13	157	2.53	Moderately pro-life
14	78	0.81	Moderately pro-life
15	134	2.13	Moderately pro-life
16	303	3.33	Strongly pro-life

^a^CDS is an acronym for cognitive distortion schemata

**Table 5:** Total CDS^a^ by category and percent of CDS^a^ by category

	Labeling and mislabeling	Catastrophizing	Dichotomous reasoning	Emotional reasoning	Disqualifying the positive	Magnification	Mental filtering	Mind reading	Fortune-telling	Overgeneralizing	Personalizing	Normative thinking
SUM	204	3	**879**	5	16	64	2	**1224**	11	36	33	**437**
%total	7%	0%	**30%**	0%	1%	2%	0%	**42%**	0%	1%	1%	**15%**

^a^CDS is an acronym for cognitive distortion schemata

### CDS by self-selected abortion identity

**Fig. 2:**
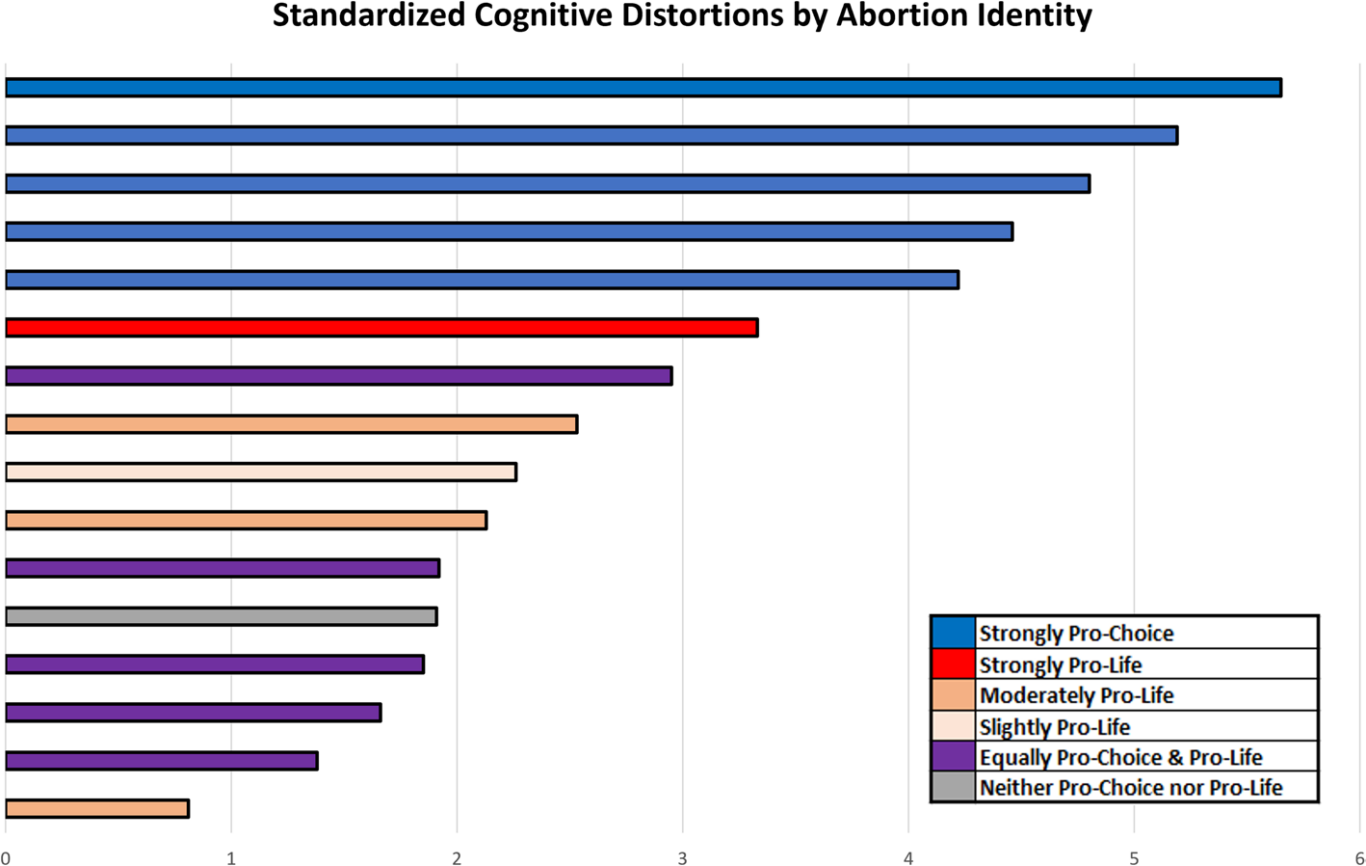
Standardized CDS per interview by abortion identity

To test whether people with polarized abortion beliefs had greater evidence of rigid thinking than those with moderate abortion beliefs, we plotted CDS per minute by abortion identity (see Fig. 2). We observed that people who identified as “strongly pro-choice” had the most observed CDS per minute, followed by the “strongly pro-life” group. People who identified as “equally pro-choice and pro-life” consistently spoke with the least amount of CDS during their interviews. However, in both the “strongly pro-choice” and “strongly pro-life” groups, we identified one outlier who spoke with fewer CDS per minute than their respective cohort and the general sample.

### LIWC summary

Table 6 displays the LIWC results, where numbers are to be interpreted as percentages. Overall, participants spoke with varying degrees of authenticity and clout and were consistently low on analytic thinking and perceptiveness. We conducted simple bivariate correlations between CDS per minute and LIWC indicators. The Pearson r coefficient of CDS by LIWC indicators is as follows: authenticity (0.71), analytic (−0.26), clout (0.33), and perceptiveness (−0.17). We acknowledge that these correlations should be interpreted with caution, given our limited sample size.

**Table 6:** Participant ID and LIWC^a^ indicators

Participant ID	Authenticity	Analytic	Clout	Perceptiveness
1	40.81	7.84	67.92	1.59
2	71.15	6.72	64.34	2.10
3	51.12	1.99	33.74	1.49
4	89.67	8.65	74.59	1.73
5	68.28	6.13	52.44	1.47
6	86.97	6.62	70.28	1.41
7	77.47	8.86	74.78	3.48
8	41.93	15.91	88.37	1.90
9	64.35	8.12	55.04	2.29
10	52.68	9.07	56.06	2.20
11	69.57	13.29	58.90	1.82
12	85.73	2.49	45.25	1.98
13	62.80	5.10	59.16	2.25
14	62.89	18.54	57.41	3.03
15	40.19	25.33	79.35	2.95
16	68.67	13.37	59.06	1.37

^a^LIWC is an acronym for Linguistic Inquiry and Word Count. Please also note numeric LIWC values are expressed as percentages

## Discussion

We examined the psychosocial properties of abortion ideology by leveraging notions of rigid and absolutist thinking central to cognitive behavioral therapy, a best practice treatment of internalizing disorders. We used NLP analyses and psychological inventories to scan qualitative interviews for cognitive distortions—markers of rigid and inflexible thinking. We hypothesized that the high prevalence of CDS might be indicative of unwaveringly firm and rigid abortion beliefs and attitudes as well as a style of thinking that is more prone to black or white reasoning. Thus, making assumptions about others’ beliefs, tendency to label, and catastrophize lower CDS prevalence may be indicative of more flexible and less rigid abortion views. Our findings provide support for such associations between CDS and belief rigidity, yet the nuance of our findings necessitates further discussion. We explain the relevance of these findings and implications for public health/opinion science below.

### The presence of CDS in interviews about abortion may denote rigid beliefs

In the USA, abortion beliefs are often dichotomized into two categories. These categories include people who support abortion rights and access, sometimes called pro-choice, and people who support restricting access to abortion in favor of fetal rights, sometimes referred to as pro-life [27]. Although many identify as either pro-life or pro-choice, a substantial portion of adults in the USA hold attitudes that are more nuanced than is captured by these labels or may be representative of both or neither of these labels. Indeed, research suggests that people’s abortion beliefs are more nuanced than such a dichotomy presupposes, as people’s attitudes tend to vary along moral and legal dimensions [2, 3, 5]. For example, an individual who leans pro-choice may identify at least one circumstance in which an abortion is not acceptable (e.g., late-term abortion or aborting if the fetus is a different gender than desired). Likewise, a person who leans pro-life may identify at least one scenario in which abortion is acceptable (e.g., if the woman’s health is endangered or if the pregnancy was a result of rape) [28].

Discrepancies or contradictions in abortion beliefs underscore the concept of abortion complexity, wherein various personal and external contexts simultaneously influence abortion beliefs and attitudes [29]. Our study assessed abortion attitude complexity in a novel way by evaluating how personal abortion beliefs are communicated in qualitative interviews about abortion (i.e., the psychology of one’s abortion beliefs). Perhaps unsurprisingly, we found that people with self-declared polarized abortion beliefs (i.e., strongly/moderately supportive of abortion or strongly/moderately antiabortion) contained more markers of cognitive distortions (CDS n-grams) in their interviews than those with temperate or uncertain beliefs. In other words, those who identified as strongly supportive of abortion or opposed to abortion communicated their views using terms and phrases that are considered cognitive distortions or markers of rigid one-dimensional thinking. Collectively, our findings suggest that people with polarized abortion views are more rigid or perhaps more unwavering in their beliefs than others with nuanced views. By contrast, participants who were more complex in their attitudes toward abortion (e.g., in the middle of a theoretical continuum of abortion attitudes) spoke with the least amount of CDS, which may infer more flexible, conflicted, or complex views. This supports both empirical notions that abortion beliefs can be strong and exist in a dichotomy. Yet, those who do not strongly associate with theoretically extreme positions on abortion may be weighing a multitude of conflicting patterns that drive the complexity of their belief systems. These findings also support a growing body of work that argues that the complexity of abortion, as a medical procedure, transcends belief systems beyond the pro-choice/pro-life dichotomy [2, 3, 5, 30].

### CDS, LIWC, and the psychology of abortion ideology

Our subsequent analysis (i.e., LIWC) substantiated findings from the CDS analysis by tying CDS usage to other psychological inventories. For example, we observed that greater CDS is tied to higher authenticity scores in the LIWC lexicon (Pearson *r* = 0.70). This suggests that those with very firm beliefs (and polarized abortion views) communicated their perspective in open, honest, and authentic terms compared with others with lower CDS prevalence. Stated differently, people with firm abortion views may express themselves with terms that allude to their passion, interest, or in-depth knowledge of the subject—which is scored as authenticity by LIWC. We contend this association (i.e., CDS and openness/honesty) may be tied to pro and antiabortion advocacy and personal passions or knowledge about abortion [31–33]. For example, people who are firm in their abortion beliefs may communicate openly and honestly because they are passionate or invested in the subject. Furthermore, people with firm beliefs may have sought sources to support those beliefs, and their language may reflect common “words of order” or “talking points” that are specifically intended to provide persuasive, clear, and unambiguous statements of their beliefs. Other studies have alluded to similar findings where polarized viewpoints point toward unwavering support or opposition for abortion, including support or opposition for telemedicine [32] and later abortions [34].

Interestingly, we also observed a (slight) inverse correlation between CDS and analytic thinking. At face value, this suggests that people with polarized abortion beliefs communicate views in language that does not denote higher-order thinking. However, it is likely that the interpretation is more nuanced. Indeed, the purpose of these interviews was to identify complexity in abortion beliefs and have people articulate how this complexity manifests in their thinking about abortion. Participants were asked a series of questions, including “How would you define abortion,” and “How does abortion make you feel?” We contend those with polarized or similarly strong views may have stated their perspective without much internal thought or deliberation. By contrast, those with “in the middle” or unknown beliefs about abortion may have deliberated much more with themselves and the interviewer, resulting in language reflected in the analytic thinking LIWC variable. Importantly, this also supports a body of research on social ideology that suggests fundamentalism—unwavering attachment to beliefs—is a strong predictor of opinions and attitudes [35]. In the future, researchers should consider a formal qualitative analysis with a larger sample to ascertain how abortion was contextualized among participants, regardless of ideology. The mixed use of qualitative and NLP methods is also highly supported in the literature [36–39].

### Frequent use of certain CDS categories may reveal how others communicate social issues

We applied the CDS lexicon to identify markers of rigid abortion beliefs. We then applied the LIWC lexicon to triangulate these findings with a range of psycholinguistic indicators from the LIWC categories. A tertiary component of this study was to examine how frequently each category of CDS was used across interviews. Surprisingly, we found an uneven use of CDS categories. Certain categories were highly represented; others were used with minimal frequency. Indeed, mindreading— *projecting one’s views to others*, dichotomous reasoning—*black or white thinking*, and normative thinking—*portraying one’s views as correct or morally right—*accounted for a combined 87% of total CDS usage. The remaining 13% of total CDS usage was dispersed among the remaining nine categories, including labeling and mislabeling—*attributing a single attribute to the whole person*, catastrophizing—*jumping to an overblown conclusion*, and disqualifying the positive—*ignoring a positive outcome by focusing on negative aspects*.

High usage of mind reading, dichotomous reasoning, and normative thinking suggests that people may express views about abortion in one-sided ways, internalize them as morally correct, and believe such views are mirrored in others. This also compliments our finding that high CDS usage, generally, is tied to rigid or firm abortion beliefs, and low CDS usage may be tied to complex beliefs. CDS categories that were not represented also inform how participants across the abortion belief system may internalize or communicate their beliefs. For example, limited use of emotional reasoning—*drawing conclusions using emotional truth over empirical evidence*—may suggest our participants did not form views based entirely on emotion. Similarly, limited use of catastrophizing—*jumping to an overblown conclusion*, may also suggest people in our sample do not view abortion urgently or hold views and beliefs about abortion that suggest a poor or terrible outcome (e.g., *Roe v. Wade* being overturned by the Supreme Court). Beyond abortion, a similar analysis of other social issues may likely reveal an overrepresentation of different CDS classes. Going forward, researchers should consider applying the CDS and LIWC lexicons for social media or qualitative data related to other social issues to compare findings with our own.

### Implications for research and policy related to abortion

Although abortion attitudes in the USA are dichotomized along pro-choice and pro-life designations, salient beliefs about abortion are actually complex, nuanced, and at times contradictory. Our pilot study sought to expand on a growing body of research on abortion complexity by underpinning how a variety of abortion beliefs are communicated via qualitative interviews. Our findings revealed that people with polarized abortion views (i.e., those who indicated strong support of or opposition to abortion) are more rigid or firm in their thinking; people with complex and nuanced views are less rigid or firm in their thinking. These findings align with public opinion literature that speaks to the political polarization in the USA and the growing ideological distance between political parties [40]. Indeed, over the last 50 years, the US electorate has slowly pulled candidates and, by extension, the government to the fringe of either party [41]. This has resulted in policies and political platforms that appeal to highly partisan constituents on either side of political preferences despite the majority of people holding beliefs on different social issues, including abortion, primarily in the middle (i.e., complexity). However, because much legislation surrounding abortion, and most other social issues, are framed along partisan lines [42], the policies governing our society may not reflect the views and beliefs of those they directly affect or be rooted in scientific evidence. More research is needed to investigate whether unbiased, scientifically driven policies are possible in a democratized society that continues to polarize.

### Limitations and adjustments for forthcoming abortion attitude research

Our study represents one portion of a pilot mixed-methods study on abortion attitudes. Findings from this study, and other subsequent studies using the same pilot data, will inform a survey and interview protocol for a national, probability-based study [43] assessing abortion beliefs in the USA. Indeed, our forthcoming study will expand on findings from this pilot investigation in several ways, including the use of a larger sample size and improved demographic representation.

First, we acknowledge an important limitation that our sample size was small relative to the number of completed surveys. We attribute the low response rate to scheduling conflicts, no-responses, no-shows, and noneffective recruitment strategies. For example, we initially contacted participants via email for participation in the follow-up interviews but realized this tactic was ineffective. As such, we modified our procedures for this study and the forthcoming study to recruit via text message, which yielded a much better response from participants.

Due to revisions made to our sampling protocol, the proposed sample will have enough participants to conduct meaningful statistical comparisons. While our pilot findings were helpful in directing us toward several *possible* associations between CDS prevalence and resultant abortion beliefs, our sample size was insufficient to perform statistical tests that would validate those findings, including anticipated comparisons across gender, race, and age groups. A larger sample size will allow us to confirm these associations by combining NLP analyses with conventional quantitative modeling. Second, our forthcoming study will be conducted in two languages [44] using probability-based sampling techniques to match current US diversity. This larger and more diverse sample size will also yield enough power for planned contrasts between and within respective groups (i.e., white vs. non-white, English vs. Spanish, and others). Both the CDS and LWIC lexicons have also been validated in Spanish. Thus, we intend to replicate our study (in two languages) given the success reported herein. Indeed, these analyses—coupled with the other findings of the larger study—will yield a more nuanced perspective of complexity in abortion attitudes, or lack thereof, among diverse groups.

## Conclusion

We applied lexical matching methods to a series of qualitative interviews to ascertain the psychological mechanics by which abortion beliefs are grounded and how beliefs are communicated to others. Based on findings from our study, we contend that people with self-identified polarized abortion identities may be more rigid in their beliefs (i.e., higher CDS prevalence) than those with moderate or uncertain abortion views (i.e., lower CDS). Furthermore, the additional use of LIWC indicators supports that high CDS usage (i.e., rigid beliefs) is also tied to authenticity or passion. Collectively, our findings suggest the extent that NLP can be leveraged to study niche aspects of opinion research.

## Data Availability

Interview transcripts cannot be shared due to constraints imposed by the Institutional Review Board.

## Abbreviations

NLP: Natural language processing
CBT: Cognitive behavioral therapy
CDS: Cognitive distortion schemata

## Footnotes

1 Our initial quota requirements included 66.7% English speaking and 33.3% Spanish speaking; 49.5%, 49.5% men, 1% transgender, 12.8% 18–24 years, 17.7% 25–34 years, 16.7% 35–44 years, 17.7 45–54 years, 16.4% 55–64 years, and 18.8% 65 and older; 25% each for Black/African American, Hispanic, White, and other; and 40% high school education or less, 30% some college or associate degree, and 30% bachelor’s, master’s, or doctoral degree.

2 GfK used a captcha item to screen for bots and a reading check item as an attention check. Participants who did not respond correctly to these items were removed from the sample.
